# Mono-ADP-ribosylation of histone 3 at arginine-117 promotes proliferation through its interaction with P300

**DOI:** 10.18632/oncotarget.20347

**Published:** 2017-08-18

**Authors:** Feng Ling, Yi Tang, Ming Li, Qing-Shu Li, Xian Li, Lian Yang, Wei Zhao, Cong-Cong Jin, Zhen Zeng, Chang Liu, Cheng-Fang Wu, Wen-Wen Chen, Xiao Lin, Ya-Lan Wang, Michael D. Threadgill

**Affiliations:** ^1^ Department of Pathology, Molecular Medicine and Cancer Research Center, Chongqing Medical University, Chongqing, China; ^2^ Department of Pharmacy and Pharmacology, University of Bath, Bath, UK

**Keywords:** site of mono-ADP-ribosylation, histone modification, colon carcinoma, proliferation, P300

## Abstract

Relatively little attention has been paid to ADP-ribosylated modifications of histones, especially to mono-ADP-ribosylation. As an increasing number of mono-ADP-ribosyltransferases have been identified in recent studies, the functions of mono-ADP-ribosylated proteins have aroused research interest. In particular, histones are substrates of some mono-ADP-ribosyltransferases and mono-ADP-ribosylated histone have been detected in physiological or pathological processes. In this research, arginine-117 (Arg-117; R-117) of hsitone3(H3) is identified as the a site of mono-ADP-ribosylation in colon carcinoma(the first such site to be identified); this posttranslational modification may promote the proliferation of colon carcinoma cells *in vitro* and *in vivo*. Using a point-mutant lentivirus transfection and using an activator of P300 allowed us to observe the mono-ADP-ribosylation at H3R117 and enhancement of the activity of P300 to up-regulate the level of acetylated β-catenin, which could increase the expression of c-myc and cyclin D1.

## INTRODUCTION

Epigenetics are widely reported to play a major role in early stages of neoplastic development and in the whole process of progression of cancer [1]. Compared with the early focus on the DNA as a critical epigenetic marks in progression of cancer, a growing body of research now pays attention to the function of histone modifications in tumourigenesis [2]. Modifications of histones include acetylation, phosphorylation, methylation, ADP-ribosylation, ubiquitylation and sumoylation, amongst which acetylation of histones has already been confirmed in the regulation of various cancers. As more mono-ADP-ribosyltransferases have been found in recent years [3–7], the function of mono-ADP-ribosylation of histones in the development of human disease, even in cancer, comes into view.

Histones are basic proteins, which bind to anionic DNA in chromosomes. In eukaryotes, there are five types of histone: H1, H2A, H2B, H3 and H4. There is strong evidence that shows that poly-ADP-ribosylation of histones plays important roles in repair and replication of DNA [8, 9] and in proliferation of cells and tumors [10]. Poly(ADP-ribosyl)ation interacts with acetylation [11], methylation [12], phosphorylation [13] of hsitones. Increasing numbers of mono-ADP-ribosyltransferases have been detected in mammals and these have the potential to catalyze the mono-ADP-ribosylation of histones. However, research data on the role of mono-ADP-ribosylation in physical or pathological process are lacking to date.

Nicotine-amide adenine dinucleotide (NAD^+^), a well-known coenzyme in redox reactions, is also an important substrate of mono-ADP-ribosylation. One ADP-ribose is transferred to specific amino-acids (arginine, histidine, cysteine and asparagine) on target proteins, in processes catalyzed by mono-ADP-ribosylatransferasees [4, 14–15]. In our previous study, we showed that changing the expression of arginine-specific mono-ADP-ribosylatransferasees affects the proliferation migration and adhesion of cells in colorectal cancer [16–18]. This suggests that mono-ADP-ribosylation plays an important role in the development of colorectal cancer. However, mono-ADP-ribosylation at different specific amino-acids sites of histones may cause different functional effects. A study of mono-ADP-ribosylation would clarify the exact mono-ADP-ribosylated amino-acid site its function in the development of cancers. We show here that H3R117 is mono-ADP-ribosylated in highly malignant colon carcinoma cells (LOVO cell line), while this does not occur in a colon carcinoma of much lower malignancy (SW480 cell line). Our research indicates that H3R117 mono-ADP-ribosylation enhances the activity of P300 to promote the proliferation of colon carcinoma.

## RESULTS

### Histone H3R117 is mono-ADP-ribosylated in highly malignant LOVO cells

**Figure 1:**
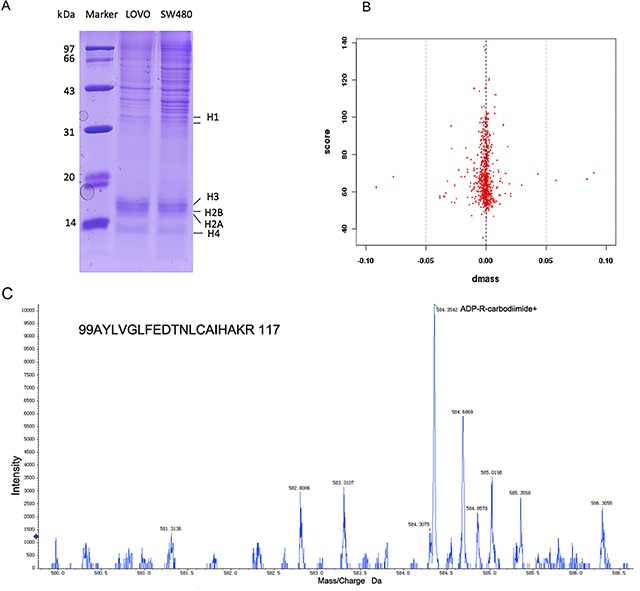
LC-MS/MS analyzed that arginine mono-ADP-ribosylation of histone **(A)** The histone of LOVO and SW480 was showed by SDSP-PAGE. **(B)** The errors distributions of confidence detected peptides. **(C)** MS/MS spectrum of the peptide precursor ions at m/z 584.4 determining mono-ADP-ribosylation at arginine 117 in the peptide of 99AYLVGLFEDTNLCAIHAKR 117 of histone H3.

The histones of LOVO cells and SW480 cells, which include H1, H2A, H2B, H3 and H4, were separated by SDPS-PAGE (Figure 1A). Normal distribution graphics of mass error distribution of peptide showedthat the fragment ions from histones of LOVO and SW480 could match simulation peptides in a data bank (Figure 1B). LC-MS/MS demonstrated that mono-ADP-ribosylation at arginine would be produced ADP-R-carbodiimide in the mass spectrometer, leading to an informative MS/MS mass spectrum (Table 1) and the number of peptides, proteins and sites where occurred mono-ADP-ribosylation occured(Table 2). MS/MS spectrum of the peptide precursor ions at *m/z* 584.4 determining mono-ADP-ribosylation at arginine 117 in the peptide of 99AYLVGLFEDTNLCAIHAKR 117 of histone H3 (Protein ID IPI00455457) which e-value is 0.0039(Figure 1C).

**Table 1:** Total second stage mass spectrum y and meaningful second stage mass spectrum

Sample	The number of Total second stage mass spectrum	The number of meaningful second stage mass spectrum	Spectrum identification rate
**SW480-T**	29209	532	1.82%
**LOVO-T**	29963	346	1.15%
**SW480-G**	24542	641	2.61%
**LOVO-G**	27189	467	1.72%

**Table 2:** The number of mono-ADP-ribosylated peptide, protein and site

Sample	Peptide NO	mono-ADP-ribosylated peptide NO	mono-ADP-ribosylated peptide Raito	Peptide NO	mono-ADP-ribosylated peptide NO	mono-ADP-ribosylated peptide Raito	mono-ADP-ribosylated site NO
**SW480-T**	382	7	1.83%	185	7	3.78%	7
**LOVO-T**	262	5	1.91%	134	5	3.73%	5
**SW480-G**	458	4	0.87%	230	4	1.74%	4
**LOVO-G**	350	6	1.71%	186	5	2.69%	6

### The construction of H3R117A and H3R117K LOVO cells

**Figure 2:**
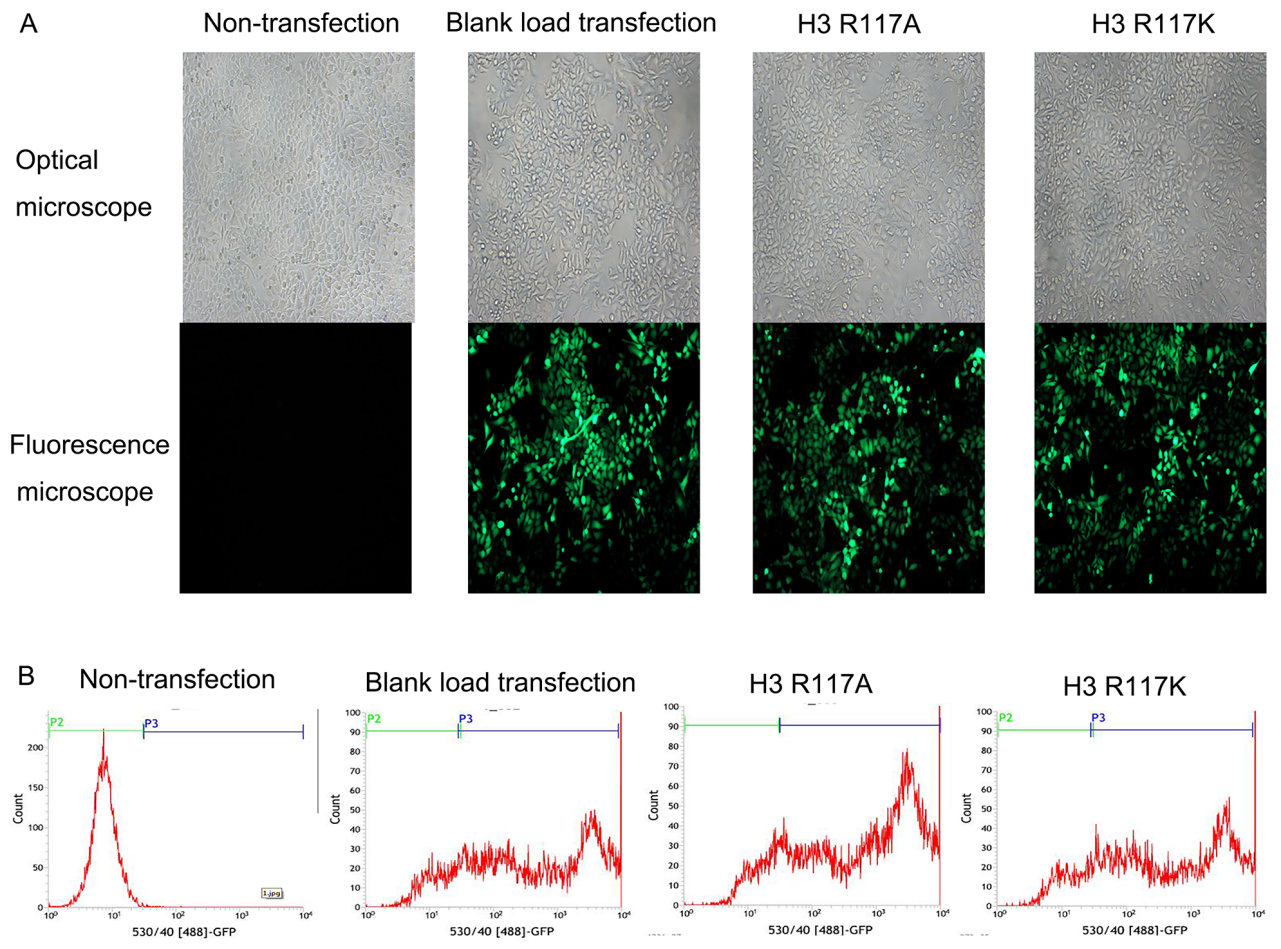
H3R117A and H3R117K LOVO cells were constructed successfully **(A)** Lentiviral infecting efficiency of LOVO cells was observed under under the microscope (× 200). **(B)** The expression rate of GFP in LOVO cells was detected by flow cytometry after transfection. Non-transfection group (P3=0.23%); Blank load transfection group (P3=76.66%); H3R117A group (P3=86.15%); H3R117K group (P3=76.69%).

PLenti-H3 mut1-IRES-Egfp-and pLenti-H3 mut2-IRES-Egfp-transfected LOVO cells were detected by the green fluorescence after the fourth day. The highest multiplicity of infection (MOI) was 20 (Figure 2A). After disposed basticidin (BSD) at the optimal concentration (4 μg/mL), the green fluorescence of H3R117A, H3R117K and blank load transfection LOVO cells were raised up to 80 % (Figure 2B). H3R117A, H3R117K, blank load transfection and non-transfection LOVO cells were prepared for follow-up experiments.

### Effects of mono-ADP-ribosylation of histone H3R117 on proliferation of LOVO cells *in vivo* and *in vitro*

**Figure 3:**
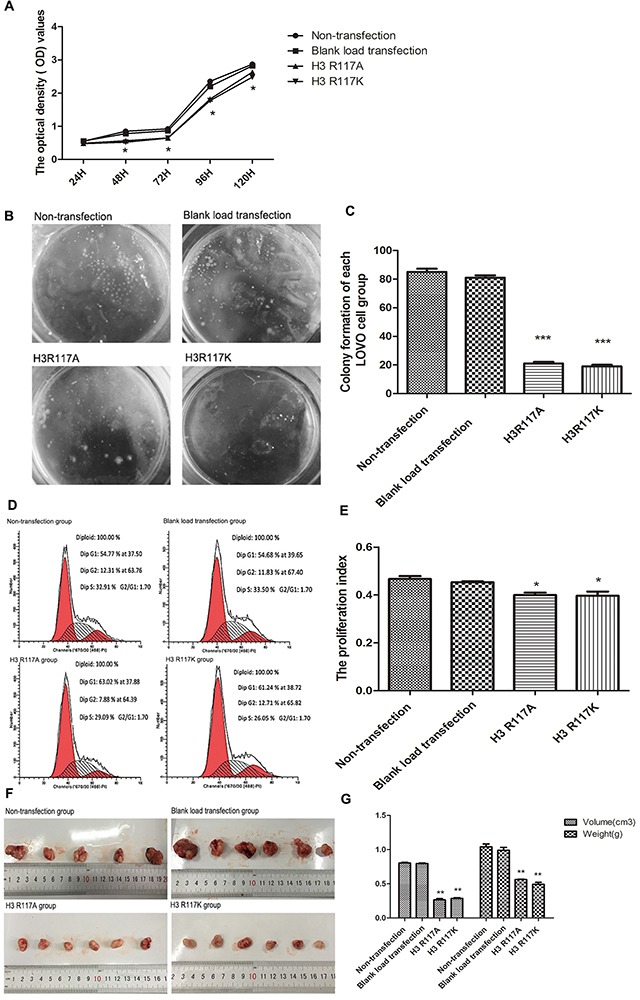
H3R117 mono-ADP-ribosylation affects the proliferation of colon carcinoma **(A)** The proliferation inhibitory rate of mutant groups increased(*p<0.05). **(B&C)** The soft agar cloning method showed that the mutant groups formed fewer cell clones, compared with the control groups(***p<0.001). **(D&E)** FCM showed that the proliferation index(PI) of the mutant groups were lower than for the control groups(*p<0.05). **(F&G)** The weight and volume of mutant LOVO cells transplanted tumors in nude mice showed a significant reduction, compared with the control groups(**p<0.01).

In order to investigate the function of H3R117 mono-ADP-ribosylation on the proliferation of LOVO cells, a cell counting kit-8 (CCK-8) assay was used to measure cell viability. This is showed the proliferation inhibitory rate of LOVO cells increased when the R117 acid of H3 was changed to alanine (A) or lysine (K) (p<0.05) (Figure 3A). The soft agar cloning method also showed that, compared with non-transfection group and blank load transfection group, the H3R117A and H3R117K groups formed fewer cell clones(p<0.001) (Figure 3B&3C). However, there is no difference between the H3R117A and H3R117K groups, indicating that the change of proliferation rate was effected by mono-ADP-ribosylation at H3R117. To test the effect further, the cell cycles in each group were assessed by flow cytometry. The proliferation index(PI) of H3R117A and H3R117K group have a decrease compared with the control groups(p<0.05) (Figure 3D&3E). H3R117 mutant LOVO cells were also transplanted subcutaneously into nude mice. the weight and volume of transplanted tumors in the H3R117A and H3R117K groups were both less than for the control nude mice which were transplanted with wild-type LOVO cells (p<0.01) (Figure 3F&3G)

### H3R117 mono-ADP-ribosylation is associated with P300 in the proliferation of LOVO cells

**Figure 4:**
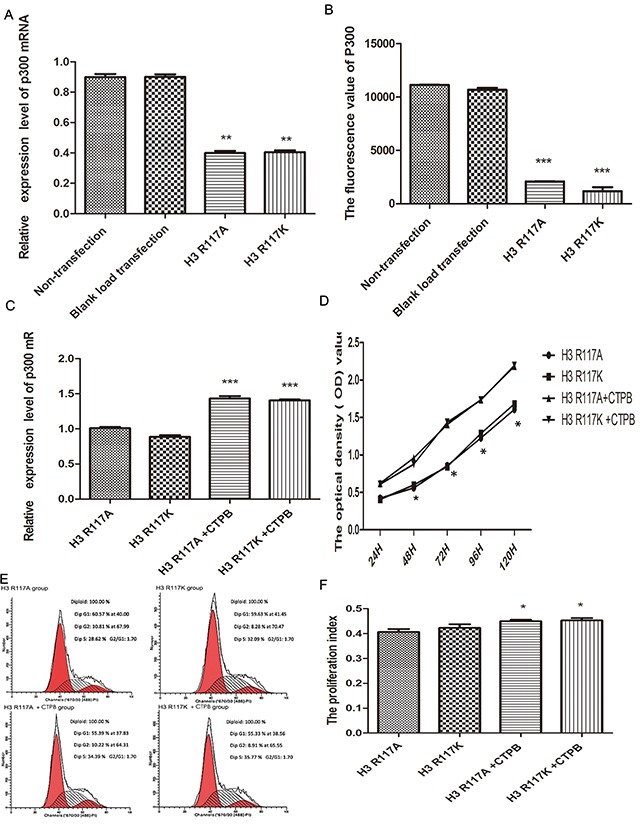
H3R117 mono-ADP-ribosylation effects on proliferation associates with P300 **(A)** The mean mRNA expression of P300 in H3R117A and H3R117K LOVO cells showed a reduction in contrast with the control groups(**p<0.01). **(B)** The P300 histone acetyltransferase activity kit detect that the activity of P300 is decreased in H3R117A and H3R117K LOVO cells(***p<0.001). **(C)** The mRNA level of P300 was raised in the mutant groups after treatment with CTPB (***p<0.001). **(D-F)** An increase of growth rate and PI in the mutant groups after treatment with CTPB (*p<0.05).

Quantitative real-time PCR (Q-PCR) was performed for detecting the mRNA of P300 between the amino-acid-mutant groups and the control groups. Compared with the non-transfection group and the blank-load transfection group, a notable and highly significant finding is that the mRNA expression of P300 in the amino-acid-mutation groups is decreased (p<0.01) (Figure 4A). However, there was no significant difference between H3R117A and H3R117K groups (p>0.05). To verify further that H3R117 mono-ADP-ribosylation could influence the histone acetyltransferase activity of P300, P300 histone acetyltransferase activity was measured. We noted that the P300 histone acetyltransferase activity is decreased in H3R117A and H3R117K groups,relative to the wild-type (p <0.001) (Figure 4B)

The effect of mono-ADP-ribosylation H3R117 on proliferation *via* P300 was confirmed further. An activator of P300 (10 μM CTBP, N-[4-chloro-3-(trifluoromethyl)phenyl]-2-ethoxy-6-pentadecylbenzamide) was used to treat the H3R117 mutant group for 24 h, and then the proliferation of LOVO cells was measured. The change of P300 mRNA level between mutant groups treated with 10UM CTPB for 24h and mutant groups without CTPB was assessed by Q-PCR assay. Compared with mutant groups without CTPB, the P300 mRNA level showed increases in the mutant groups treated with CTPB (p<0.001), which illustrates the CTPB could increase the amount of P300 protein in mutant LOVO cells. Correspondingly, there was no significant difference between these two mutant groups treated with CTPB(p>0.05) (Figure 4C). Then, to test whether the proliferation of H3R117A and H3R117K LOVO cells lacking the mono-ADP-ribosylated modification on H3R117 could be regulated by the activity of P300, a CCK8 assay was performed. These results showed an increase of growth rate in the mutant groups treated with CTPB compared to the mutant groups without CTPB(P<0.05) (Figure 4D). FCM also showed that the PI of mutant groups was increased after treating with CTPB(p<0.05) (Figure 4E&4F). Together, these results suggest that mono-ADP-ribosylation at H3R117 regulates the proliferation of LOVO cells, possibly through P300.

### Adjustment of H3R117 mono-ADP-ribosylation or P300 affect the expression c-myc and cyclinD1 proteins *in vivo* and *in vitro*

**Figure 5:**
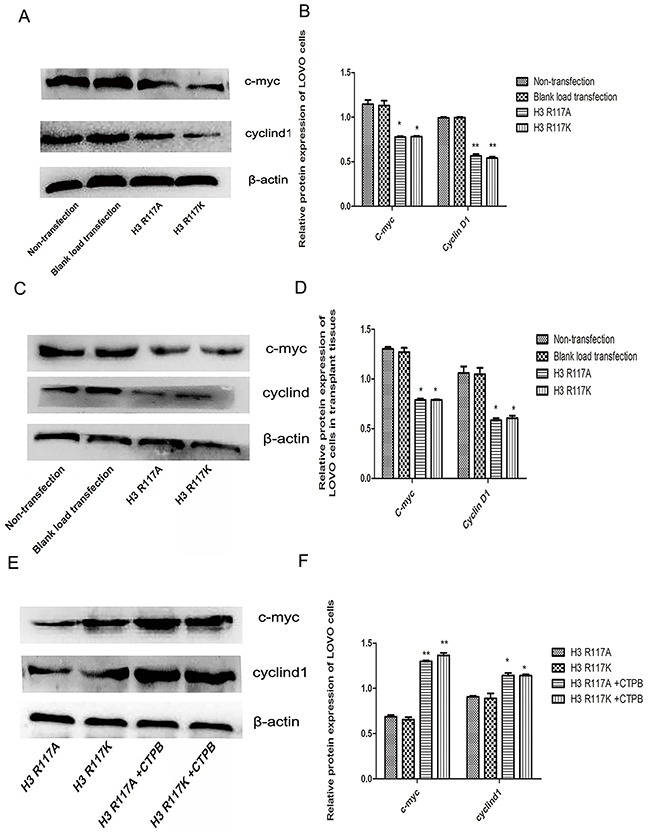
Adjustment of H3R117 mono-ADP-ribosylation or P300 affects β-catenin, c-myc and cyclinD1 **(A & B)** Effects on the change of protein expression level in each LOVO cells group. Expression of c-myc and cyclin D1 in the LOVO cells groups showed a significant decrease in the mutant groups compared with the control LOVO groups (*p<0.05, ** p<0.01). **(C & D)** Proteins were extracted from nude mouse transplanted tumor tissues. The results of protein expression *in vitro* were in accordance with *in vivo* (*P<0.05). **(E&F)** Western blots showed that the expressions of c-myc and cyclinD1 in the mutants treated with CTBP groups were significantly increased compared with the mutant groups without CTBP (*p<0.05,** p<0.01).

**Figure 6:**
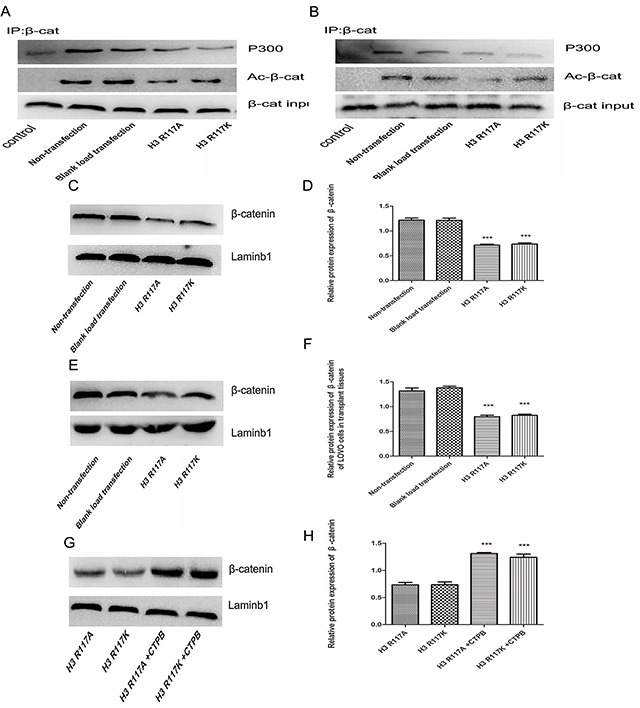
Adjustment of H3R117 mono-ADP-ribosylation or P300 affect β-catenin and the interaction of P300 and acetylation β-catenin **(A&B)** Effects on the interaction of P300 and acetylation β-catenin between the mutant groups and the control groups *in vivo* or *in vitro*. Western blot analysis was conducted on the β-catenin immune complex with specific anti-acetylated lysine antibody. β-catenin was immunoprecipitated from each LOVO cells as an input by Western blot. **(C-H)** The changes of β-catenin expression in H3R117 mutant groups and in mutant groups with CTBP or not (***p<0.001).

The mechanism by which mono-ADP-ribosylation at H3R117 could regulate the proliferation of LOVO cells, in connection with P300, was unclear. Western blotting experiments showed that, both *in vivo* and *in vitro*, the expressions of proliferation-related regulators (c-myc, cyclinD) in the H3R117A and H3R117K group were lower than in the control groups (p<0.05) (Figure 5A-5D). Correspondlingly, treatment of the H3R117A and H3R117K groups with the P300 activatorCTPB to caused expressions of c-myc and cyclinD to be increased (Figure 5E&5F). Mono-ADP-ribosylation at H3R117 possibly regulates these proliferation-related regulators through P300. It is known that P300 would catalyze acetylation of β-catenin, which could affect the expression of important proliferation-related proteins c-myc and cyclinD1. A co-immunoprecipitation assay was adopted to detect how the H3R117 mono-ADP-ribosylation affects the interaction between p300 and β-catenin. The change of H3R117 mono-ADP-ribosylation could affects the connection between acetylated β-catenin and P300 (Figure 6A&6B). Both *in vivo* and *in vitro*, the expression of β-catenin decreased in the H3R117A and H3R117K groups (P<0.05) (Figure 6C-6F). After treatment with the P300 activator CTPB, the expression of β-catenin also was increased in the H3R117A and H3R117K groups (Figure 6G&6H). Hence, mono-ADP-ribosylation at H3R117 may regulate the activity of P300 in catalyzing the acetylation of β-catenin and thus affect the amount of β-catenin protein.

## DISCUSSION

Post-translational mechanism is a vital process in modification of proteins,with consequent regulation of the activity or function of the target proteins. Mono-ADP-ribosylation is one post-translational modifications involved in regulation of development of colorectal cancer. Arginine-specific mono-ADP-ribosyltransferase1(ART1), one of several mono-ADP-ribosyltransferase, has been reported to play an important role in various diseases [19–22]. Our previous studies have shown that ART1 is associated with apoptosis, proliferation and migration of colon carcinoma CT26 cells [19, 23, 24]. In addition, ART1 is associated with angiogenesis in human colorectal carcinoma tissues [25]. We knew that mono-ADP-ribosylation promotes the development of colon carcinoma, although the specific mono-ADP-ribosylatied amino acid site and target protein was not clear. This is indeed the challenge in studying the detailed effects of this post-translational modification and its influence on signaling pathways. Moreover, it contributes to provide a theoretical basis for the therapeutic approaches against various diseases [26–28]. We employed LC-MS/MS to analyze the site of mono-ADP-ribosylated modification on histone3 in different malignant cell lines and were pleased to find that this histone is mono-ADP-ribosylated only in the highly malignant colon carcinoma cell line (LOVO cells). One of these sites is H3R117. To detect the function of mono-ADP-ribosylation of H3R117 in colon carcinoma, we changed the arginine to other amino-acids (lysine or alanine); the mono-ADP-ribosylation is specific to arginine as the target amino-acid. Effects on the proliferation of LOVO cells were observed. Previous research had shown that the levels of mono-ADP-ribosylated histones changed in mouse mastocytoma P815 and human chronic myelogenous leukemia K562 cells treated with 5 mM butyrate or serum-free media to block proliferation. Furthermore, it is suggested that the mono-ADP-ribosylation is the first stage of the poly-ADP-ribosylated histones which have been detected in rapidly dividing cells [29]. Mono-ADP-ribosylated histone may be an important regulative factor in cellular proliferation. In present study, the data showed that the cell growth inhibition ratio increased and the proliferation index reduced in H3R117A and H3R117 modified LOVO cells; Moreover, the weight and volume of tumors derived from these H3R117A and H3R117 LOVO cells after subcutaneous transplantation in nude mice were both decreased. These results indicate that arginine-specific mono-ADP-ribosylation of H3R117 might regulate the proliferation of LOVO cells.

Several studies have shown that different types of post-translational modification of histones not only affect the interactions with the proteins, but also could interplay mutually. Studies have shown that, when an acetylation of a histone is detected, it is occasionally accompanied by mono-ADP ribosylation modification which suggests an interaction between acetylation and mono-ADP ribosylation of histones. However, the mechanism needed to be elucidated. The transcriptional co-activators CREB binding protein (CBP) and p300 are members of the histone acetyltransferase(HAT) family. CBP is highly homologous with P300. CBP and P300 have been reported to acetylate histones and non-histone proteins, which result in different effects on gene transcription [30–35]. The effects of P300 have been proven to be involved in cell cycle regulation, cell differentiation and cell apoptosis [36, 37]. Activated poly-(ADP-ribose) polymerase 1 (PARP1) can upregulate the activity of acetyltransferase of P300 [38]. In our previous studies, both arginine-specific mono-ADP-ribosyltransferase1 (ART1) and poly-(ADP-ribose) polymerase 1 (PARP1) were shown to promote the development of colon carcinoma (CRC). PARP-1 is affected by regulation of ART1 through NF-κB [39]. In addition, there is a synergistic effect of ATR1 and PARP-1 on the apoptosis induced by cisplatin in CT26 cells. PARP-1 was found downstream of ART1, which suggests PARP-1 activity may be modulated by ART1. Additionally, the histone mono-ADP-ribosylation reaction is the basis of the poly-(ADP-ribosyl)ation rections which regulate the cell proliferation [40]. Accordingly, we speculate whether the mono-ADP-ribosylation at H3R117 could affect P300. We observed that the production of mRNA and the histone acetyltransferase activity of P300 in the H3R117 mutation groups is decreased. Hence, H3R117 mono-ADP-ribosylation may be connected with P300.

Moreover, studies have reported that P300 can interplay with β-catenin and the interaction between P300 and β-catenin makes influences Wnt signaling pathway in regulation of the cell physiology, including the proliferation, differentiation and apoptosis of colon carcinoma(CRC) cells [41–45]. In addition, in colon carcinoma, the interaction of P300 with β-catenin has important impacts on the development of colon cancer cells [46–48]. Therefore, we investigated whether mono-ADP-ribosylation at H3R117 promotes proliferation of LOVO cells *via* P300. We added an activator of P300 (CTBP) into H3R117A and H3R117K LOVO cells to re-activate the P300 and observed that the proliferation index of H3R117A and H3R117K LOVO cells both increased. This is a preliminary indication that mono-ADP-ribosylation at H3R117 dosed enhance the proliferation of LOVO cells *via* P300.

Studies have also reported that P300 can acetylate β-catenin to enhance the transcription of β-catenin and promote the combination of β-catenin with transcription factor 4 (TCF4), resulting in activating the Wnt/β-catenin signaling pathway [43, 45, 46, 48]. β-catenin acts as a vital part of the Wnt pathway, through activating the signaling pathway resulting in various human diseases including cancer. It is reported extensively that the Wnt pathway regulates growth, proliferation and apoptosis of cells. C-myc and cyclin D1 are downstream of the Wnt pathway relating to the proliferation of cancer cells. P300 acetylates β-catenin at lysine 49, which is reported frequently in cancer. Importantly, mutation of this site lead to an activation of c-myc gene through the augmentatiion of β-catenin [46]. In addition, β-catenin regulates the expression of cyclinD1 in colon carcinoma cells [49]. In the present study, our results are consistent with these reports. The expression of β-catenin, c-myc and cyclin D1 was diminished in the H3R117A and H3R117K groups, which implies that the mono-ADP-ribosylation at H3R117 has an effect on up-regulating the expression of β-catenin, c-myc and cyclin D1, the vital proliferation relative proteins. However, the specific mechanisms of these need to be further determined. To elaborate the mechanisms in more detail, we also added CTPB to H3R117 mutant LOVO groups and observed that the expression of β-catenin, c-myc and cyclin D1 were all increased. These results point towards the conclusion that H3R117 mono-ADP-ribosylation may increase the proliferation-related proteins β-catenin, c-myc and cyclin D1 *via* P300.

In addition, to verify how H3R117 mono-ADP-ribosylation impacts the combination of the P300 and β-catenin, a co-immunoprecipitation assay was showed how change of H3R117 mono-ADP-ribosylation could affect the connection between acetylated β-catenin, which detected by anti-acetyl-lysine antibody, and P300. It has been reported that P300 interacts with β-catenin in multiprotein complexes which could catalyzed acetylation of β-catenin. An anti-acetyl-lysine Western blot is more sensitive for detecting β-catenin acetylation [46, 50]. In addition, because acetylation of β-catenin can enhance the transcription of β-catenin, increase in acetylation of β-catenin could enhance the expression of β-catenin. We also noted that the expression of β-catenin decreases in the H3R117 mutant LOVO groups. Addition of CTBP into these cells led to increase in the expression of of β-catenin. This indicates that H3R117 mono-ADP-ribosylation influences the proliferation proteins c-myc and cyclin D1 through the acetylation of β-catenin by P300.

In our study, we detect firstly the site of mono-ADP-ribosylation on H3 in colon carcinoma cells and focus on the function of arginine site of mono-ADP-ribosylation modification in the proliferation of these cells. In addition, we study the possible molecular mechanisms in detail; it appears that H3R117 mono-ADP-ribosylation enhances the activity of acetyltransferase P300 activity towards β-catenin, leading to activation of downstream genes (c-myc, cyclin D1). However, the detail of how H3R117 mono-ADP-ribosylation affects P300 remains to be studied. Nevertheless, our findings on the role of H3R117 mono-ADP-ribosylation on colon carcinoma may lead to identification of a target for individualized treatment of colon cancer.

## MATERIALS AND METHODS

### Cell lines, reagents and animals

Human colon adenocarcinoma LOVO cells line and SW480 cells line obtained kind gift from Professor Wei-Xue Tang, Chongqing Medical University (Chongqing, China). They were cultured in dulbecco's modified eagle medium (DMEM) medium (Hyclone, Logan, UT, USA) with 10 % fetal bovine serum(Hyclone) under the conditions of 5 % CO^2^ and 37°C. 10 μM CTPB [N-(4-chloro- 3-trifluoromethyl-phenyl)-2-ethoxy-6-pentadecyl-benzamide] (Santa Cruz Biotechnology, Santa Cruz, CA, USA) was added to the cells for 24h. Nude mice were provided by the Experimental Animal Center of National Bio-industry Base in Chongqing Medical University.

### LC-MS/MS screening the mono-ADP-ribosylatied site on histones

This study was completed by the BGI-Huada Genomics Institute (Shenzhen, China). Cultured cells (5×10^6^ cells/ mL) in 1.5 mL tubes. The method of Schechter was used for extraction of histones [51]. The supernatant was removed carefully with a pipette and the histone pellet was air-dried for 20 min at room temperature. The pellet was dissolved in an appropriate volume of double-distilled H2O (typically 100 mL). Histones were stored frozen at 80°C before use the concretion of histones was assayed by the Bradford method. The histones from LOVO and SW480 cells were separated on a 12% SDS-PAGE gel and stained with Coomassie Brilliant Blue solution. 50 ug of histone from LOVO or SW480 was denatured by 8 M urea and then processed by reductive alkylation(DTT/IAM). The sample was diluted until the concentration of urea was diminished to 2 M and each sample was divided into two parts. The one part, which was labelled SW480-T or LOVO-T, was added trypsin (trypsin/protein =1:100) and the samples were incubated for 5 min at 37°C. 0.5% formic acid was added to terminate the reaction. To the other part, which was labelled SW480-G or LOVO-G, was added Glu-C (Glu-C: trypsin=1:100) and the mixture was incubated for 20 min overnight. Again, 0.5% formic acid was added to terminate the reaction. The derived mixture of peptides was desalted with C_18_ column, dried by vacuum-pumping and re-dissolved with 20 μL 0.1% formic acid. The quality was controlled by Matrix-Assisted Laser Desorption Ionization (MALDI) mass spectrometry and the peptides were then detected by LC-MS/MS (TripleTOF™ 5600, AB SCIEX, Boston, MA, USA). The software Mascot v2.3 was used to search the protein and the site of arginine mono-ADP-ribosylation in the mass spectral database IPI human v3.87. The parameters are as follows:

**Table:** 

Item	value
**Type of search**	MS/MS Ion search
**Enzyme**	SemiTrypsin
**Fragment Mass Tolerance**	±0.1Da
**Mass Values**	Monoisotopic
**Variable modifications**	Oxidation (M), Methyl (K), Arg->Orn ®, Acetyl (K)
**Peptide Mass Tolerance**	±0.05Da
**Fixed modifications**	Carbamidometh®®
**Instrument Type**	ESI-QUAD-TOF
**Database**	IPI human (91464 sequences)

### Construction of point-mutated H3R117 LOVO cells

The H3R117 point mutation sequence was designed and synthesized by Geneart (Regensburg, Germany). The sequence to change the H3 Arg-117 to alanine(H3R117A) is as follows: GGATCCaccatggcccgtactaagcagactgcccgcaagtcgaccggcggcaaggccccgaggaagcagctggctaccaaagcggcccgcaagagcgcgccggccacgggcggggtgaagaagccgcaccgctaccggcccggcaccgtggctctgcgggagatccggcgctatcagaagtctacggagctgctgatccgcaagctgcccttccagcggctggtacgcgagatcgcgcaggagtttaagacggacctgcgcttccagagctcggccgtgatggcgctgcaggaggccagagaggcctacctggtggggctgttcgaagacacgaacctgtgcgccatccatgccaagGCCgtgaccatcatgcccaaggacatccagttggtcagccgcatccgcggggagcgggcctgaGGCGCGCC. The sequence of change the H3 117^th^ arginine to lysine(H3R117K) is as follows: GGATCCaccatggcccgtactaagcagactgcccgcaagtcgaccggcggcaaggccccgaggaagcagctggctaccaaagcggcccgcaagagcgcgccggccacgggcggggtgaagaagccgcaccgctaccggcccggcaccgtggctctgcgggagatccggcgctatcagaagtctacggagctgctgatccgcaagctgcccttccagcggctggtacgcgagatcgcgcaggagtttaagacggacctgcgcttccagagctcggccgtgatggcgctgcaggaggccagagaggcctacctggtggggctgttcgaagacacgaacctgtgcgccatccatgccaagAAAgtgaccatcatgcccaaggacatccagttggtcagccgcatccgcggggagcgggcctgaGGCGCGCC. The two sequences were respectively wrapped with pLenti6.3_MCS_IREs2-EGFP and named pLenti-H3 mut1-Ires-EGFP or pLenti-H3 mut2-Ires-EGFP, which were constructed by Invitrogen (Shanghai, China). LOVO cells were transfected with pLenti-H3 mut1-Ires-EGFP and pLenti-H3 mut2-Ires-EGFP to construct H3R117A and H3R117K site-directed mutation LOVO cells.

### Cell counting kit-8 (CCK-8) assay

100 μL of LOVO cells (5×10^3^ cells per well) were dispensed in 96-well plates and respectively incubated for 24 h, 48 h, 72 h, 96 h, 120 h under appropriate conditions (5 % CO^2^, 37°C). After 24 h,48 h, 72 h, 96 h or 120 h, respectively. 10 μL CCK-8(Dojindo, Kumamoto, Japan) solution was added in each well respectively and the mixtures were incubated for 1 h. The absorbance (optical density, OD) was read with a universal micro plate reader (BioTek, Winooski, VT, USA) at 450nm. The percentage of growth inhibition rate was calculated by the formula: [1-(mean OD of experimental group – mean OD of blank control group)/(mean OD of Non-transfection group – mean OD of blank control group)] ×100%.

### Soft agar cloning method

1.2 % Agarose (Invitrogen, Carlsbad, CA, USA) and 0.7% agarose were used, respectively, as upper stratum and underlay. The 10^4^ cells in each group were treated with supplemented with culture medium (10% fetal bovine serum and pre-warmed DMEM medium). Then the above cells were plated in 60mm dishes. Specifically, before the cells were seeded in the plates, an equal volume of 1.2% argorse contained was added to the plates. When the underlay agarose solidified at room temperature, the above cells was seeded in plates in a mixture of 0.7 % argorse and culture medium. Then each plate was incubated in incubator (37°C, 5 % CO_2_) for 15 d. The clones (approximately 50 cells in each clone) were identified and counted under the microscope (DM3000, Leica, Solms, Germany).

### Flow cytometry analysis of cell cycle

Cells (1 × 10^4^) of each group were collected into centrifugal tubes, washed twice with PBS, suspended with the culture medium and centrifuged at 1000 rpm for 5 min. Cells were then washed with 70 % ethanol and centrifuged at 1000 rpm for 5 min for flow cytometry (FACS Vantage SE, Becton Dickinson and Company, Franklin Lakes, NJ, USA). The proliferation index(PI) was measured according to: PI=(G2+S) /(G1+S+G2).

### Construction of the tumor transplantation model

LOVO cells (1×10^7^/mL) collected from each group were injected into armpits of nude mice. Nude mice were placed in the Specific Pathogen-Free(SFP) feeding room (20-26°C, 12 h dim and 12 h light) of the animal experimental center in Chongqing Medical University. After 2 weeks, mice were weighted and sacrificed. Then the transplant subcutaneous sarcomas were harvested and the tissue proteins were exacted from these excised tumors for detection of the expression of proteins of interest.

### Quantitative real-time PCR

Quantitative real-time PCR was used to determine the relative expression levels of P300 MRNA. RNA was extracted according to the manual for the Trizol reagent (Takara, Dalian, China). Then, complementary DNA was synthesized with the directions of the PrimeScriptTMRT reagent Kit with GDNA Eraser (Perfect Real Time) (Takara, Dalian, China). The GAPDH gene was used as reference to normalize the expression of the transcript. The primers were designed by Sangon Biotech Co (Shanghai, China), as follows: P300, 5′-TTCAAACGCCGAGTC TTCTT-3′(F) and 5′-GCTGCTGTTGGCATAGG3′(R); GAPD,5′-GGTGAACGCTTGAACG-3′(F) and 5′-TGTTAGTGGGGTCTCC-3′(R). Each 25 μL PCR master mix included 2.0 μL CDNA template (<100 ng), 1.0 μL forward primer (10 μM),1.0 μL reverse primer (10 μM), 12.5 μL SYBR Premix Ex Taq and 8.5 μl RNase-free H_2_O. The subsequent quantitative real-time PCR followed the directions of the SYBR Premix Ex TaqTM(Takara, Dalian, China) as follows:: pre-denaturation at 95°C for 30 s, denaturation 40 cycles at 95°C for 5s, annealing at 58°C for 20 s and elongation at 72°C for 20 s. The amplification efficiency in each sample was greater than 99%.2^−△△Ct^ and the absolute value of the slope in each sample was greater than 0.1.

### Histone acetyltransferase assay

A kit which used fluorescence for quantitative measurement of the histone acetyltransferase activity of P300 (GenMed scientific inc, Minneapolis, MN, USA) was used. The assay was followed according to the manufacturer's protocol. The fluorescence intensity value of each group was measured by the fluorescence microplate reader.

### Western blot analysis

The total or nuclear proteins of cells and nude mouse transplantation tumors of the colorectal cancers were extracted using a total and nuclear protein extraction kit (Beyotime, Shanghai, China). The concentration of protein was detected with a BCA protein assay kit (Beyotime). Then, proteins were separated by a SDS-PAGE gel and transferred to Poly (vinylidene fluoride)(PVDF) membranes (Millipore, MA, USA) by electrophoresis. The membranes were blocked with 5% non-fat milk for 1 h and incubated with primary antibodies against c-myc (Santa Cruz Biotechnology), cyclinD1(Proteintech Group, Chicago, IL, USA), β-actin (Bioss, Shanghai, China) at 4°C overnight respectively. Peroxidase-conjugated goat anti-rabbit IgG (Bioss) was used to incubate membranes for 2 h at room temperature. Finally, the membranes were assessed using enhanced chemiluminescence reagents (ECL) (Beyotime) and analyzed with Quantity One software.

### Co-immunoprecipitation

Lysates from each group were generated with the Piece Co-IP Kit (Thermo Scientific, Bonn, Germany). Then the co-immunoprecipitation (Co-IP) was carried out according to the manufacturer's instructions. Briefly, the rabbit polyclonal β-catenin antibody (Cell Signaling Technology, Danvers, MA, USA) was first immobilized for 2 h onto Amino Link Plus coupling resin. The resin was then washed and incubated with lysate overnight at 4°C. Subsequently, the resin was washed again and then eluted by elution buffer. Elution buffers were collected and analyzed by Western blotting which used rabbit polyclonal β-catenin antibody(Cell Signaling Technology, Danvers, MA,USA), rabbit polyclonal p300 antibody antibody (Santa Cruz Biotechnology), rabbit polyclonal acetylated-lysine antibody(Cell Signaling Technology) and peroxidase-conjugated goat anti-rabbit IgG (Bioss). A negative control was employed in the Co-IP kit to assess non-specific binding.

### Statistical analysis

Experiments were replicated at least thrice. Values were presented as mean ± standard deviation (SD) (x ± s). One-way ANOVA or Student's t-test, which were processed by SPSS 19.0 software (SPSS, Chicago, IL, USA), were used to analyze the statistical significance between each group. A p-value less than 0.05 indicated a statistically significant difference.

## Abbreviations

ART: ADP-ribosyltransferases
ART1: Arginine-specific mono-ADP-ribosyltranferase1
Arg;R: Arginine
A: Alanine
K: Lysine
H3: Histone 3
BSD: Blasticidin
PARP1: Poly(ADP-ribose) polymerase 1
NAD^+^: Nicotine-amide adenine dinucleotide
CTBP: N-[4-Chloro-3-(trifluoromethyl)phenyl]-2-ethoxy-6-pentadecylbenzamide
ADP: Adenosine diphosphate
CBP: CREB-binding protein
CRC: Colon carcinoma
Q-PCR: Quantitative real-time PCR
CCK-8: Cell counting kit-8
ECL: Enhanced chemiluminescence
PVDF: Polyvinylidene difluoride
Co-IP: Co-immunoprecipitation
SFP: Specific Pathogen Free
SDPS-PAGE: Sodiumdodecylsulfate polyacrylamide gel electrophoresis
TCF4: transcription factor 4
MOI: Multiplicity of infection
OD: Optical density
(SD) (x ± s): Mean ± standard deviation
